# Validity of the International Physical Activity Questionnaire (IPAQ) for assessing moderate-to-vigorous physical activity and sedentary behaviour of older adults in the United Kingdom

**DOI:** 10.1186/s12874-018-0642-3

**Published:** 2018-12-22

**Authors:** Claire Cleland, Sara Ferguson, Geraint Ellis, Ruth F. Hunter

**Affiliations:** 1 UKCRC Centre of Excellence for Public Health (NI)/Centre for Public Health, Queen’s University Belfast, Institute for Clinical Sciences B, Royal Victoria Hospital, Grosvenor Road, Belfast, UK; 2 School of Natural and Built Environment, Queen’s University Belfast, David Keir Building, Belfast, UK

**Keywords:** Physical activity, Moderate-to-vigorous physical activity, MVPA, Sedentary behaviour, Validity, International physical activity questionnaire, IPAQ, Self-report, Accelerometry, Objective measurement

## Abstract

**Background:**

In order to accurately measure and monitor levels of moderate-to-vigorous physical activity (MVPA) and sedentary behaviour (SB) in older adults, cost efficient and valid instruments are required. To date, the International Physical Activity Questionnaire (IPAQ) has not been validated with older adults (aged 60 years plus) in the United Kingdom. The current study aimed to test the validity of the IPAQ in a group of older adults for both MVPA and SB.

**Methods:**

Participants wore an Actigraph GT3X+ for seven consecutive days and following the monitor wear participants were asked to complete the IPAQ. Statistical analysis included: Kolmogorov-Smirnov tests; descriptive analyses; Spearman’s rho coefficients; and Bland-Altman analyses.

**Results:**

A sample of 253 older adults were recruited (mean age 71.8 years (SD 6.6) and 57% male). In total, 226 had valid accelerometer and IPAQ data for MVPA and 228 had valid data for SB. Results showed the IPAQ had moderate/acceptable levels of validity (*r* = .430–.557) for MVPA. For SB, there was substantial levels of validity on weekdays (*r* = .702) and fair levels of validity (*r* = .257) on weekend days. Bland-Altman analysis showed inherent measurement error with the majority of participants tending to under-report both MVPA and SB. Results showed the majority of older adult’s under-report their level of MVPA and SB when completing the IPAQ and the linear relationship above the mean shows an error from under to over reporting as the mean increases.

**Conclusions:**

Findings from the current study suggest that the IPAQ is better implemented in larger surveillance studies comparing groups within or between countries rather than on an individual basis. Findings also suggest that the IPAQ validity scores could be strengthened by providing additional detail of types of activities older adults might do on a daily basis, improving recall; and it may also be necessary to provide an example of a daily break down of typical activities performed. This may enable older adults to more fully comprehend the amount of time they may spend active, sitting and/or lying during waking hours.

## Background

Numerous urgent calls to action have been made to combat the global physical inactivity ‘pandemic’ [1]. Given the rapidly ageing nature of our society, there is a specific need to focus future research on the physical activity behaviours of older adults [2]. However, previous research has demonstrated that measurement of these behaviours is “fraught with challenges” [3, 4], with measurement error a particular issue. It is important that this programme of future research include studies to develop and validate measures of physical activity and sedentary behaviour for older adults that are accessible and useable by researchers and practitioners. However, physical activity measurement in older adult populations is difficult as it has to ensure that it accounts for the differing physical and psychological characteristics of this population including physical functioning and cognitive decline [5–8].

Measurement of physical activity and sedentary behaviour in older adults can be performed in various ways, including the implementation of subjective (indirect) and objective (direct) instruments [4]. The implementation of indirect subjective measurement relies heavily upon the individual and their ability to self-report their level of physical activity and sedentary behaviour over a period of up to seven days through the completion of a questionnaire. This type of measurement approach provides researchers with an inexpensive, efficient and simple method, placing only a low level of burden on the participant and research team [4]. However, indirect subjective measurement is often subject to biases as it relies on the older adult’s cognitive function and memory recall, and can pose issues regarding reading/vision difficulties [9–11]. Limitations of subjective measurement of physical activity and sedentary behaviour have been well-documented, and also include desirability bias [6, 12, 13].

An alternative to subjective measurement is the use of objective tools (direct) such as accelerometers, pedometers or combined monitors. It has been reported that such direct measures provide increased accuracy as they do not rely on self-report and recall bias. However, as they require up to seven days’ of wear by the participant, and a research team with the expertise and time to initiate the monitors, implement the study and to process the data they may not be the most feasible method of physical activity measurement [14].

As the type, intensity and metabolic cost of physical activities vary for older adults, subjective measurement approaches may provide the required level of detail, and overcome the methodological inconsistencies that have been found with objective measurement tools [4]. In addition, subjective measurement tools may be considered a more cost efficient and practical alternative as they only require a short period of time to complete in comparison to a seven-day period of wear [14]. However, in order to make between or within group, city or country comparisons in large scale studies of older adults, it is essential that subjective physical activity measurement tools are valid and reliable.

The International Physical Activity Questionnaire (IPAQ) is a commonly used measurement tool. Designed as a standardised self-report questionnaire, IPAQ can provide researchers and practitioners with an estimate of physical activity and sedentary behaviour for adults aged 15–69 years, across a range of socio-economic settings [15–17]. Moreover, the IPAQ is also beneficial for researchers collaborating within, or between countries at differing sites [15, 18]. Currently, the IPAQ is under-explored regarding its ability to measure sedentary behaviour, and in particular, within an older adult sample. Previous research has focused on validity of the IPAQ in adult populations [19, 20]. Further, previous research has shown the IPAQ to be a valid tool for the measurement of physical activity and sedentary behaviour in older adults in Belgium (moderate validity, *r* = 0.33–0.40) [20], Japan (adequate validity, *r* = 0.42–0.53) [21]; and Hong Kong (acceptable reliability and validity, *r* = 0.47) [19]. However, the IPAQ has yet to be validated for an older adult population (60 years or older) within the United Kingdom, or for those aged 70 years and above [16].

Therefore, the aim of the current study was: 1) to assess the validity of the IPAQ (long-form) when measuring moderate-to-vigorous physical activity; and 2) to assess the validity of the IPAQ (long-form) when measuring sedentary behaviour, in an older adult population in the United Kingdom (UK) (compared to the Actigraph GT3X).

## Methods

### Sample recruitment

The current study is a sub-study of the wider Healthy Urban Living and Ageing in Place (HULAP) Project [22]. Participants were recruited for the HULAP Project from a sub-sample of older adults from Wave One of the Northern Ireland Cohort for the Longitudinal Study of Ageing (NICOLA) (aged 60 years plus) (http://nicola.qub.ac.uk/). The NICOLA Study is Northern Ireland’s first long-term study of ageing, involving 8500 men and women aged 50 years and over. Participants in the NICOLA Study were randomly selected from across Northern Ireland, and are representative of the Northern Ireland population (https://www.qub.ac.uk/sites/NICOLA/FileStore/Filetoupload,783215,en.pdf).

Eligibility criteria for the HULAP Project and subsequently the current study included: completion of the NICOLA Study (computer assisted personal interviewing (CAPI)); agreement to be re-contacted to participate in follow up research studies; aged 60 years and above; self-reported ability to walk 10 m unassisted; and ability to provide written informed consent to participate in the study. Potential participants who were selected for recruitment were initially sent a letter of invitation and a study information sheet. As a follow up to the postal invitation, participants were contacted approximately 1 week later by a study researcher (CC or SF), via telephone, in order to discuss their potential participation in the current study.

### Measures of physical activity

Older adults, aged 60 years plus, in the UK (the two largest cities in Northern Ireland - Belfast and Londonderry) were asked to wear an accelerometer for a period of seven consecutive days, and to subsequently complete an IPAQ (long-form). Participants who agreed to participate received a study pack including: a study instruction sheet; consent forms; an accelerometer wear instruction sheet; a monitor wear time diary; a questionnaire (which included the IPAQ (long-form)); and a tri-axial accelerometer (Actigraph GT3X+, Actigraph Inc., Florida, US).

### IPAQ (long-form)

Following the seven-day period of accelerometer wear, participants were asked to complete a study questionnaire which included questions regarding their demographic characteristics (gender, age, ethnicity, nationality, highest educational attainment, relationship status and current situation) and the IPAQ (long-form). The IPAQ (long-form) consists of 27 questions which reflect on the previous 7 days’ activities according to domain: 1) occupational physical activity; 2) transportation physical activity; 3) housework, house maintenance and caring for family; 4) recreation, sport and leisure-time physical activity; and 5) time spent sitting [23].

### IPAQ (long-form) processing

The IPAQ data was entered manually by CC into SPSS Data Analysis Version 23 (SPSS Inc., Chicago, IL). Ten percent of data was then checked for accuracy (by RH) of entry, of which results showed 100% accuracy. All IPAQ data was cleaned and processed by CC using the standardised IPAQ Scoring Protocol [16].

### Accelerometry

The Actigraph GT3X+ (Actigraph Inc., Florida, US) is a small, lightweight, and unobtrusive device that measures acceleration in three planes (vertical, horizontal front to back, and horizontal left to right). For the purposes of this study, it was set to record acceleration data 30 times every second (30 Hz) and participants wore the device on an elasticated belt around the waist, placed on the midline of the right hip over the course of a seven-day period. This enables comparative analysis to be performed with the retrospective 7 day IPAQ (long-form). Participants were asked to wear the monitor during waking hours, except when bathing, swimming or doing any other water-based activity, and to complete the wear time diary for the same period of seven consecutive days. The Actigraph GT3X+ has been validated against doubly labelled water, indirect calorimetry and oxygen consumption, and implemented in numerous validation studies as the reference for subjective measurement tools [15, 24–26].

### Accelerometer processing

Raw accelerometer activity counts were processed in Actilife 6 (Actigraph Inc., Florida, US). All activity that was recorded at 30 Hz was processed and the raw data was aggregated to 15-s epochs. The criteria used in the current study was guided by previous research [2, 27–29], specifically for older adults. The processing criteria implemented in this study was: 1) 120 min of ‘non-wear’ time with periods of 120 min zeros allowing for 2 min ‘spikes’ of activity which were less than 100 counts per minute; 2) a valid day was defined as a 24-h period in which more than 600 min of wear time was recorded; 3) participants were required to wear their monitor for at least 5 days (including one weekend day) to be considered a valid week; and 4) the following cut-points were applied to the data to categorize different intensities: sedentary (≤ 99 counts min^− 1^), light (101–1041 counts min^− 1^), MVPA (> 1042 counts min^− 1^) [27]. This set of cut points were chosen for the current study as they were established through laboratory walking tests in a healthy sample of older adults (64–77 years) [27]. The threshold was set for moderate physical activity at ≥1041 counts per minute due to a mean V0_2_ of 13 ml·kg^− 1^·min^− 1^, at a walking speed of 3.2 km/hr. which is equivalent to 3.7 METS [27].

The accelerometer data was processed using ActiLife 6 (Actigraph Inc., Florida, US) and exported to Microsoft Excel in .csv format. Within Microsoft Excel, minutes of sedentary behaviour were calculated as ‘mean minutes per week day’ and ‘weekend day’; and moderate-to-vigorous physical activity was calculated as ‘mean minutes per week’ matching that of the IPAQ outcome variable. Minutes of moderate-to-vigorous physical activity per week was determined by participants having a minimum of 5 valid days of wear and following the calculation below. For the purpose of the current study accelerometer data were calculated as continuous variables.

$$ \mathrm{Minutes}\ \mathrm{of}\ \mathrm{MVPA}/\mathrm{week}=\left(\mathrm{total}\ \mathrm{MVPA}\ \left(\mathrm{minutes}\right)/\mathrm{number}\ \mathrm{of}\ \mathrm{valid}\ \mathrm{days}\right)\ \mathrm{x}\ 7\ \left(\mathrm{days}\right) $$

### Statistical analysis

It should be noted that a power analysis was not calculated for the current study. Statistical analysis was performed using SPSS Data Analysis Version 23 (SPSS Inc., Chicago, IL). In the first instance, tests for normality were performed by implementing Kolmogorov-Smirnov tests. Descriptive analyses were then performed on the demographic variables of the sample. Due to the non-normal distribution of the IPAQ and accelerometer data, median, inter-quartile ranges (IQR) and non-parametric tests were performed.

Differences in the self-report (IPAQ (long-form)) versus objective (accelerometer) measures were assessed using Wilcoxon-signed rank tests for: 1) minutes of moderate-to-vigorous physical activity per week; 2) minutes of sedentary behaviour per week day; and 3) minutes of sedentary behaviour per weekend day.

Spearman’s rho coefficients were performed to determine the association and the bivariate correlation coefficients between each method (self-report versus objective) for both moderate-to-vigorous physical activity and sedentary behaviour. Bivariate correlations were also performed by gender and age categories. To interpret the Spearman’s rho coefficient, we used the following benchmarks: 0–0.20 = poor correlation, 0.21–0.40 = fair correlation, 0.41–0.60 = moderate/acceptable correlation, 0.61–0.80 = substantial correlation, and 0.81–1.0 = near perfect correlation [30].

Finally, Bland-Altman analyses determined the level of agreement for moderate-to-vigorous physical activity (MVPA) per week, minutes of sedentary behaviour per week day and minutes of sedentary behaviour per weekend day [31]. This analysis is a method used to determine how closely two methods that can be used to measure the same outcome are in agreement and the degree of concordance [32]. Bland-Altman analyses were performed for both moderate-to-vigorous physical activity and sedentary behaviour (week day and weekend day). The following formulas were used:

- 1a. Mean = ([IPAQ minutes of MVPA per week + accelerometer minutes of MVPA per week]/2) and;
- 1b. Difference = [IPAQ minutes of MVPA per week - accelerometer minutes of MVPA per week].
- 2a. Mean = ([IPAQ minutes of sedentary behaviour per week day + accelerometer minutes of sedentary behaviour per week day]/2) and;
- 2b. Difference = [IPAQ minutes of sedentary behaviour per week day - accelerometer minutes of sedentary behaviour per week day].
- 3a. Mean = ([IPAQ minutes of sedentary behaviour per weekend day + accelerometer minutes of sedentary behaviour per weekend day]/2) and;
- 3b. Difference = [IPAQ minutes of sedentary behaviour per weekend day - accelerometer minutes of sedentary behaviour per weekend day].

Limits of agreement were calculated as follows: mean difference between instruments (IPAQ minus accelerometer) ± (1.96 × standard deviation). Significance was determined at the level of *p* < 0.05.

## Results

**Fig. 1:**
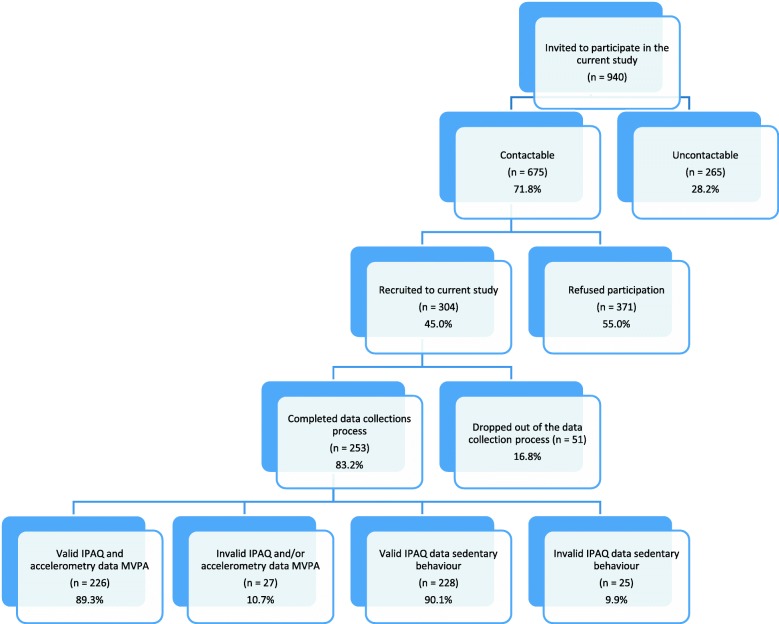
Flow Diagram of Participant Recruitment

Of the individuals who were invited to participate, 675 of 940 (71.8%) were contactable (Fig. 1). The research team were not able to contact the remaining 28.2% due to disconnected phone lines, call guardian, and failure of potential participants to return voicemails. Of those participants that could be contacted, the recruitment rate was 45.0% (*n* = 304), and the retention rate of those who agreed to participate in the study was 83.2% (*n* = 253). Reasons for non-retention included; illness (*n* = 15), illness of a partner (*n* = 11), lack of time (*n* = 12) and no reason given (*n* = 13); no demographic information was retained for these participants so bias analysis was not performed. Of the 253 individuals who participated in the study, 89.3% (*n* = 226) had both valid accelerometer and IPAQ data meeting applied criteria for MVPA, and 90.1% (*n* = 228) for sedentary behaviour (Fig. 1).

### Demographic characteristics

The majority of participants (*n* = 226) in the study were aged 60–70 years (46.9%, *n* = 106, mean age of 71.8 years (SD 6.6)), male (57.1%, *n* = 129); white (98.2%, *n* = 222); British (54.9%, *n* = 124), married (66.4%, *n* = 150), with high school level education (GCSE/O-Levels/Intermediate/Junior Cert) or equivalent (20.8%, *n* = 47); and retired (81.9%, *n* = 185) (Table 1).

**Table 1:** Demographic characteristics of the study sample

		Overall sample n
Gender	*Male*	129
	*Female*	97
Age (years)	*60–70*	106
	*71–80*	85
	*81–90*	18
	*91 plus*	4
Ethnicity	*White*	222
Nationality	*British*	124
	*Irish*	36
	*Northern Irish*	58
	*English*	2
	*Scottish*	3
	*Other*	1
Relationship status	*Married*	150
	*Living with a partner*	2
	*Single*	20
	*Separated*	4
	*Divorced*	14
	*Widowed*	34
Highest educational attainment	*Primary school (not complete)*	5
	*Primary or equivalent*	15
	*High School (GCSE/O-Level/Intermediate/Junior*	47
	*Cert)*	29
	*High School (A-Level/Leaving Cert)*	36
	*Diploma/Certificate*	39
	*Undergraduate primary degree*	44
	*Postgraduate/higher degree*	4
	*None*	4
Current situation	*Retired*	185
	*Employed*	17
	*Self-employed*	7
	*Permanently disabled or sick*	8
	*Looking after home or family*	3
	*Other*	3

### Assessment of moderate-to-vigorous physical activity

Compared to accelerometry derived MVPA (median of 1291.0 min per week; IQR 917.3–1642.8), participants under-estimated their self-reported IPAQ derived levels of MVPA (median of 965 min per week; IQR 340.0–1785.0) (Table 2). Wilcoxon Signed Ranks Test showed that there was a significant *(p <* .001*)* difference between the self-report and objective measures for minutes of MVPA per week. For Spearman’s Rank correlation, results showed that the correlation coefficient was moderate/acceptable *r* = .52 (significant at the 0.01 level (2-tailed)) (Table 2).

**Table 2:** Analysis of moderate-to-vigorous physical activity data as measured by IPAQ and accelerometer

Physical activity measurement tool	Median minutes of MVPA per week (SD)	Inter quartile range	Wilcoxon Signed Ranks Test	Spearman’s Rank Correlations
IPAQ	965.0 (1064.6)	340.0–1785.0	0.001	.52^a^
Accelerometer	1291.0 (488.7)	917.3–1642.8		
Males				
IPAQ	780.0 (1049.2)	347.5–1575.0	0.011	.56^a^
Accelerometer	1174.0 (469.1)	798.0–1495.0		
Females				
IPAQ	1140.0 (1081.7)	326.3–1867.5	0.024	.43^a^
Accelerometer	1371.8 (481.3)	1132.6–1750.1		

^a^Correlation is significant at the 0.01 level (2-tailed)

When analyses were performed by gender, both males and females under self-reported their level of MVPA. Males reported a median of 780 min (IQR 347.5–1575.0) of MVPA per week, whereas the accelerometer recorded a median of 1174.0 min (IQR 798.0–1495.0). Females reported a median of 1140.0 (IQR 326.3–1867.5) using the IPAQ, whereas the accelerometer recorded a median of 1371.8 (IQR 1132.6–1750.1) minutes of MVPA per week (Table 2). Wilcoxon Signed Ranks Tests showed significant differences between both measures for males and females (*p <* 0.05); and Spearman’s Rank Correlations were found to be moderate/acceptable for male’s *r* = .56 and female’s *r* = .43 (Table 2) (both significant at the level of 0.01 level (2-tailed)).

### Assessment of sedentary behaviour

Results showed that both the full and gender-stratified sample underestimated their level of sedentary behaviour for both week days and weekend days (Table 3). The median time spent sedentary was 300.0 min/day (IQR 197.5–420.0) for weekdays and 300.0 min/day (IQR 240.0–420.0) for weekend days using the IPAQ. This was significantly (*p* < 0.005) underestimated as objective accelerometry measures reported a median sedentary time of 486.9 min/day on weekdays (IQR 425.8–566.5) and 501.4 min/day (IQR 436.2–580.2) on weekend days; equating to a difference of approximately three hours (Table 3).

**Table 3:** Analysis of sedentary behaviour data as measured by IPAQ and accelerometer

Physical activity measurement tool	Median minutes of sedentary behaviour per day (SD)	Inter quartile range	Wilcoxon Signed Ranks Test	Spearman’s Rank Correlations
WEEK DAY				
IPAQ	300.0 (161.1)	197.5–420.0	.000	.70^a^
Accelerometer	486.9 (99.1)	425.8–566.5		
Males				
IPAQ	300.0 (159.5)	240.0–435.0	.000	.49^a^
Accelerometer	513.5 (96.3)	460.5–600.8		
Females				
IPAQ	240.0 (156.4)	180.0–360.0	.000	.47^a^
Accelerometer	453.2 (88.8)	393.0–522.5		
WEEKEND DAY				
IPAQ	300.0 (153.8)	240.0–420.0	.000	.26
Accelerometer	501.4 (103.7)	436.2–580.2		
Males				
IPAQ	360.0 (149.2)	240.0–480.0	.000	.55^a^
Accelerometer	529.3 (98.5)	459.3–597.2		
Females				
IPAQ	240.0 (154.6)	180.0–360.0	.000	.36^a^
Accelerometer	477.1 (103.0)	419.9–549.7		

^a^Correlation is significant at the 0.01 level (2-tailed)

When results were presented by gender, the same pattern of underestimation for sedentary time existed. Both males and females underestimated their sedentary time on both week and weekend days (Table 3). Males underestimated this behaviour by 213.5 min on a weekday (300.0 min (IQR 240.0–435.0) using the IPAQ versus 513.5 min (IQR 460.5–600.8) measured by the accelerometer), and by 169.3 min on a weekend day (360.0 (IQR 240.0–480.0) using the IPAQ versus 529.3 min (IQR 459.3–597.2) (Table 3) by the accelerometer). Females under reported sedentary behaviour by 213.2 min on weekdays (240.0 min (IQR 18.0–360.0) using the IPAQ versus 453.2 min (IQR 393.0–522.5) using the accelerometer), and by 223.1 min on weekend days (255.0 min (IQR 180.0–360.0) using IPAQ versus 477.1 min (IQR 419.9–549.7) as measured by the accelerometer). This underestimation equates to a difference of approximately three and a half hours for both genders (Table 3).

For both the full and gender-stratified sample, Wilcoxon Signed Ranks Tests showed significant differences between measures (*p <* 0.05). Spearman’s Rank Correlations were found to be moderate/acceptable overall for weekdays (*r* = .70), and fair overall for weekend days (*r* = .26). Furthermore, results showed moderate/acceptable for males on both weekdays (*r* = .49) and weekend days (*r* = .55); and moderate/acceptable for females on week days (*r* = .47), and fair on weekend days (*r* = .36) (Table 3).

### Assessment by age category

For minutes of MVPA per week, results showed that those who were classified as ‘old’ (aged 60–79 years) had a higher Spearman’s correlation coefficient of r = .49 compared to *r* = .46 for those classified as ‘oldest old’ (aged 80 years and over). In contrast, for sedentary behaviour those who were classified as ‘oldest old’ had higher correlation coefficients *r* = .57 (week day) and *r* = .73 (weekend day) compared to the ‘old’ group (r = .46 (week day) and *r* = .45 (weekend day)).

### Bland-Altman agreement

**Fig. 2:**
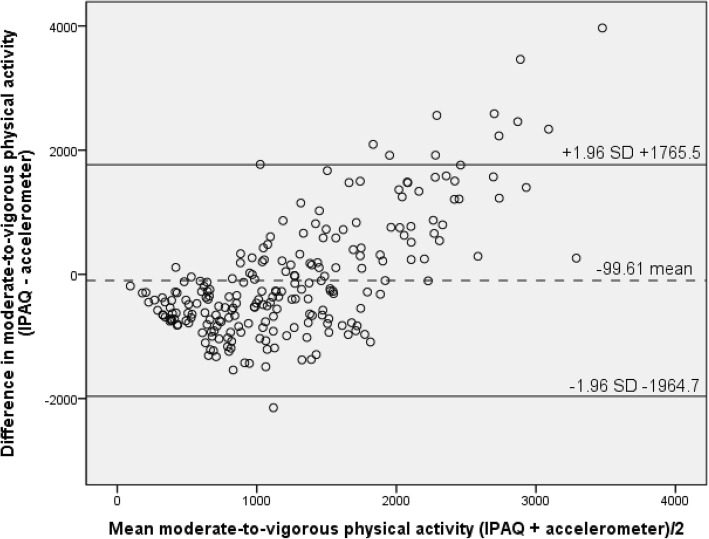
Bland-Altman plot for moderate-to-vigorous physical activity

For MVPA, results from Bland-Altman analyses highlighted a mean difference of − 99.61 min of MVPA per week (SD 951.6) between the IPAQ and accelerometer data. The limits of agreement were wide, with the difference between 1765.5 and − 1964.7 min/day (Fig. 2). The Bland-Altman Plot (Fig. 2) suggests the presence of a measurement bias, as the majority of points on the scatterplot graph fall and cluster below the mean difference and zero line. This suggests that for the majority of older adults within the current sample under-reporting of their level of MVPA is an issue when completing the IPAQ (Fig. 2). In addition, the Bland-Altman analysis also showed the linear relationship that can be seen above the mean line suggests that those older adults who are very active over-report using the IPAQ; and an error can be seen from under to over reporting as the mean increases (Fig. 2).

**Fig. 3:**
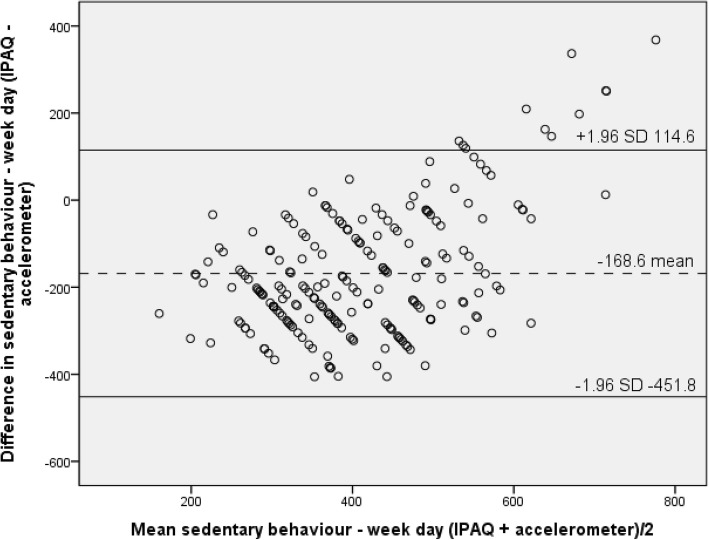
Bland-Altman plot for sedentary behaviour – week day

**Fig. 4:**
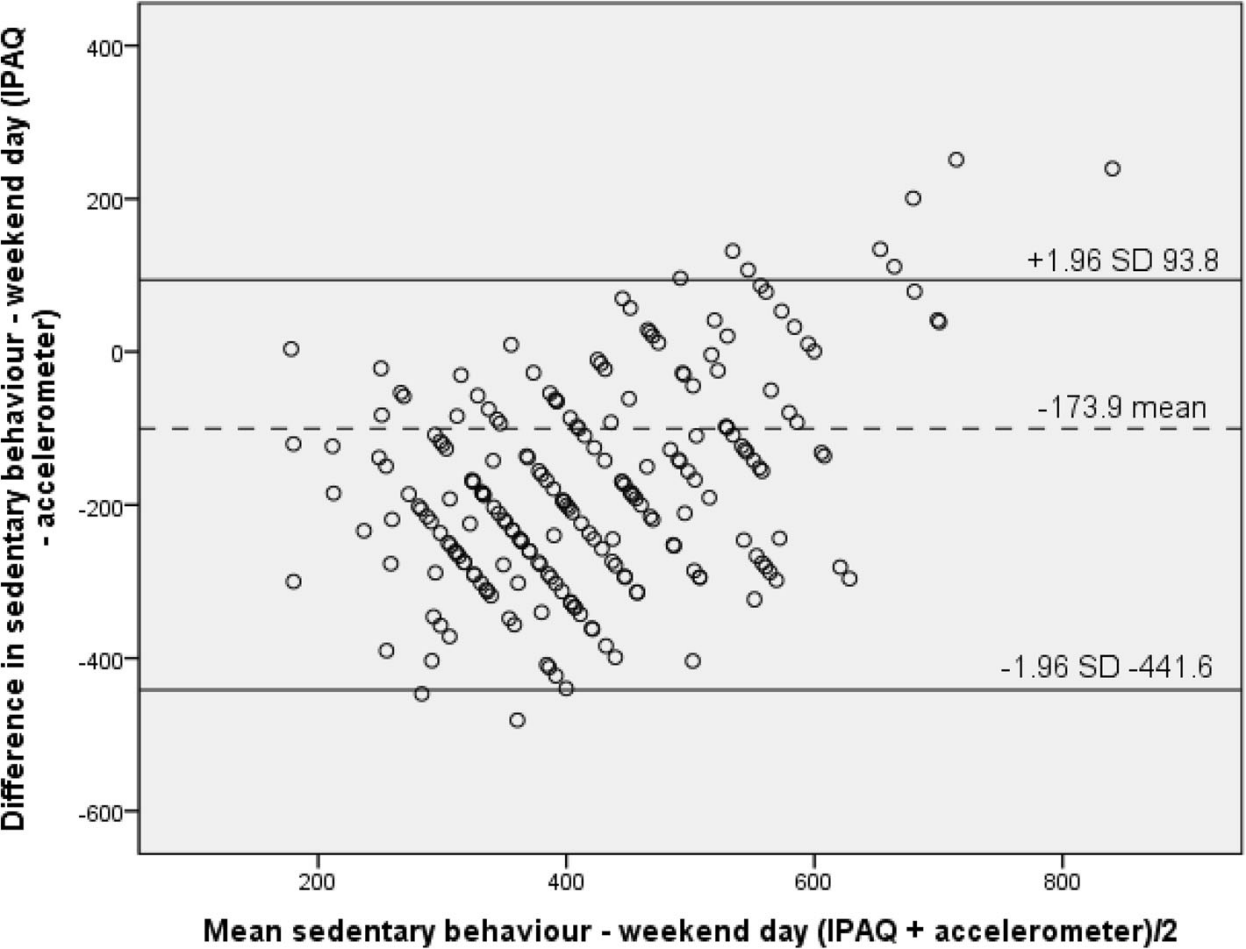
Bland-Altman plot for sedentary behaviour – weekend day

For sedentary behaviour, the mean difference between the IPAQ and accelerometer data was − 168.6 min per day (SD 144.5) during weekdays, and − 173.9 min per day (SD 136.6) for weekend days. Again the limits of agreement were wide, with the difference for sedentary behaviour on weekdays between 114.6 and − 451.8 min per day (Fig. 3) and between 93.8 and − 441.6 min per day (Fig. 4) on weekend days. Similar to the results presented for moderate-to-vigorous physical activity, both plots for sedentary behaviour indicated evidence of measurement biases, as the majority of points within the plot fall below the mean and zero line. Both the plots (Figs. 3 and 4) suggest that the majority of older adult’s under-report both their week day and weekend day sedentary behaviour with the IPAQ. Furthermore, for those individuals who are sedentary for a large proportion of their day they over-reported their sedentary behaviour when using the IPAQ; and an error can be seen from under to over reporting as the mean increases (Figs. 3 and 4).

## Discussion

The aim of this study was to determine the validity of the IPAQ (long-form) for the measurement of MVPA and sedentary behaviour when compared with Actigraph GT3X accelerometer measurement for older adults (60 years and older) in the UK. When considering the findings from the current study it should be noted that the cut points implemented to determine minutes of MVPA (≥1041 counts per minute) were established in a laboratory setting by Copeland & Esliger (2009) in a sample of healthy living older adults (64–77 years) [27]. The cut points may appear to be low and to overestimate levels of physical activity via accelerometry and consequently finding show an underestimation by the IPAQ. However, we feel this is not the case as the cut points were calibrated in a laboratory setting (treadmill walking test - 3.2 km/hr) and found a mean V0_2_ of 13 ml·kg^− 1^·min^− 1^, which is equivalent to 3.7 METs [27]. On review of the Compendium of Physical Activities 3–6 METs is considered as a moderate physical activity; therefore, the established cut point of ≥1041 counts per minute would be a “conservative delineation of MVPA for older adults” [27, 33, 34]. In addition, the work by both Kwan et al., (2004) and Freedson et al., (1998) supports the use of Copeland & Esliger (2009) cut points for older adults [27, 35, 36]. With Freedson and colleagues (1998) highlighting that whilst the sample of older adults in the Copeland & Esliger (2009) study may have been walking at a lower treadmill speed the energy cost to both study groups were the same, demonstrating that energy cost increases with age and lower cut points are required for MVPA [27, 36].

### Validity of the IPAQ for moderate-to-vigorous physical activity

Results found moderate/acceptable levels of validity when measuring levels of MVPA recorded using the IPAQ compared with accelerometer measurements. Spearman’s correlations showed a range of *r* = .43–.56 for the overall sample, and for both genders, which suggests a moderate/acceptable level of validity [30]. These results are comparable to previous similar research by Van Holle et al., (2015), who found a correlation coefficient of *r* = .40 for a sample of older adults in Belgium [20]. A correlation coefficient of *r* = .51 was also found for total IPAQ physical activity versus accelerometer measured light to vigorous physical activity within a validity study of the IPAQ (long-form) with Chinese elders [19]. Further, results from the current study found higher correlations (r = .43–.56 versus *r* = .18–.24) with accelerometer data for IPAQ (long form) compared to a study carried out by Grimm et al., 2012 who validate the IPAQ (short form) [37]. With regards to similar questionnaires measuring MVPA, the Global Physical Activity Questionnaire was also found to have a moderate/acceptable level of validity within a sample of adults with mean age of 44 years [30, 38].

Results from the current study showed that correlation statistics demonstrated moderately/acceptable validity for measuring physical activity and sedentary behaviour in older adults, although findings from the Bland-Altman analyses indicate inherent measurement error (as suggested by the wide confidence intervals). Results showed the majority of older adult’s under-report their level of MVPA when completing the IPAQ and the linear relationship above the mean shows an error from under to over reporting as the mean increases. This would suggest that when the IPAQ (long-form) is implemented on an individual-level basis, the validity would be somewhat reduced in comparison to when it is implemented in larger surveillance studies comparing groups within or between countries. This finding has been previously reported in other validation studies of self-report physical activity measures; with the GPAQ (adults) and IPAQ (adults) both being reported to be less accurate at the level of the individual [38, 39].

In order to implement this questionnaire in an older adult sample, it may be possible to further strengthen the validity scores by providing additional detail of the types of activities older adults may do. This may improve their ability to recall their activity over the course of a seven-day period; a problem highlighted by Prince et al., (2008) within their review of self-report validation studies [40]. Previous research has shown that this is an issue with older adult populations, as it can be challenging to recall physical activities (particularly higher intensity activities) as they perform these in an unstructured manner during their daily lives (house work, gardening etc) [41, 42]. This differs when measuring activity in younger adults who are more likely to participate in specific quantifiable physical activities such as: a sixty-minute fitness class or a ninety-minute football match. Research by Heesch et al., (2010) supports this assumption as they reported older adults finding completion of the IPAQ challenging due to difficulties with; understanding the word ‘usually’; using bouts of 10 min; and having to quantify their activity by frequency, intensity and duration [42]. Furthermore, the IPAQ (long-form) does not capture light intensity physical activities which have been shown in previous research to have important health benefits for older adults [43, 44].

### Validity of the IPAQ (long-form) for sedentary behaviour

Results from the current study demonstrated fair to substantial (*r* = .26–.70) validity for sedentary behaviour reported on weekend and weekdays respectively, and also for males and females. The difference between weekend and week day may be due to the fact that weekend days are often less structured than week days and are more difficult to quantify resulting in only fair correlations. In terms of results for Bland-Altman analyses sedentary behaviour also indicated evidence of measurement biases similar to moderate-to-vigorous physical activity; with the majority of points falling below the mean and zero line. Both the plots for week days and weekend days suggest that older adult’s under-report sedentary behaviour when completing the IPAQ; and an error appears from under to over reporting as the mean increases. The results from the current and similar studies that have aimed to validate the IPAQ have been found to show consistently higher correlations with accelerometry than validations of similar self-report measures such as the GPAQ for sedentary behaviour [38, 45].

Larger IQRs were found for sedentary behaviour when measured by the IPAQ (long-form) in comparison to the accelerometer. This further strengthens the argument that the IPAQ (long-form) may not be a useful tool to use on an individual basis when aiming to measure sedentary behaviour in older adults. Nevertheless, it is moderate/acceptable when used in large population studies.

It is accepted that questionnaires such as the IPAQ need to be brief in order to reduce participant burden. However, when they are implemented within an older adult population it may be appropriate to add further detail which can enable older adults to better understand what is meant by each question, particularly regarding sedentary behaviour. It may also be necessary to provide an example of a daily break down of typical activities performed. This may enable older adults to more fully comprehend the amount of time they may spend sitting and/or lying during waking hours. This is in line with recommendations from Heesch et al., (2010) who suggested the addition of relevant examples to provide clarity [42]. It should also be noted that sedentary behaviour is unlike MVPA in the sense that even though it carries the burden of memory recall regarding underreporting, it also carries the challenges associated with social desirability.

### Strengths and limitations

Strengths of the current study included a representative sample of older adults in the UK and concordant measurement of physical activity and sedentary behaviour using the IPAQ and accelerometer for a period of seven consecutive days. In terms of limitations of the current study, when aiming to validate a subjective measurement of physical activity, doubly labelled water (DLW) would be considered the gold standard for energy expenditure [46]. However, DLW as a measurement tool is not only expensive to implement but it also requires professional expertise making it unfeasible for most research groups to implement [24]. Therefore, a limitation of the current study is the fact that an alternative method of validation was implemented; accelerometry. Nevertheless, it should be noted that previous research has shown that accelerometry provides an acceptable and feasible measure of physical activity and sedentary behaviour in place of DLW [24, 46]. Accelerometers are commercially available and provide a relatively inexpensive and easy to implement method of measurement in a study of free living participant’s. However, it should be noted that accelerometers do have limitations which should be considered not only in the context of the current study but also for other accelerometer studies. Firstly, depending on the processing criteria (runs of zeroes, number of valid hours in a day, number of valid days in a week and the cut-points) that have been selected and implemented by the research team this will ultimately have an impact on the resultant minutes of MVPA and sedentary behaviour. This is a limitation of all accelerometer studies and when deciding specific criterion to implement, researchers should review previous work in the field and determine the best way of processing their data. Within the current study as previously stated we reviewed the work of Copeland & Esliger (2009) in older adults and felt this specific groups of cut points would be the best to implement in order to process our accelerometry data [27]. Secondly, in addition, to data processing stage of accelerometry during data collection phase accelerometers also have the limitations of failing to measure non-ambulatory activities such as weight lifting or cycling and they cannot be used to measure water based activities such as swimming or water aerobics.

## Conclusions

Results from the current study suggest that the IPAQ has moderate/acceptable validity for measuring moderate-to-vigorous physical activity for both genders of older adults in the UK. It was also found to have a substantial level of validity for sedentary behaviour week days, and fair validity for sedentary behaviour for weekend days for both genders of older adults in the UK. Furthermore, as measurement error and suggested under/over-reporting was found it would be recommended that the IPAQ (long form) is adapted for older adults (60 years and older) in order to provide further clarification on what is meant by each question. By doing so, researchers will reduce bias by assisting older adults with recall and aim to prevent social desirability, consequently improving the accuracy of this self-report measure.

## Abbreviations

CC: Claire Cleland
DLW: Doubly Labelled Water
GE: Geraint Ellis
GPAQ: Global Physical Activity Questionnaire
IPAQ: International Physical Activity Questionnaire
IQR: Inter-Quartile Range
MVPA: Moderate-to-Vigorous Physical Activity
RH: Ruth Hunter
SB: Sedentary Behaviour
SD: Standard Deviation
SF: Sara Ferguson
UK: United Kingdom
